# Survival Benefits of Adjuvant Chemotherapy for Positive Soft Tissue Surgical Margins Following Radical Cystectomy in Bladder Cancer with Extravesical Extension

**DOI:** 10.3390/curroncol30030245

**Published:** 2023-03-10

**Authors:** Prithvi B. Murthy, Shreyas Naidu, Facundo Davaro, Philippe E. Spiess, Logan Zemp, Michael Poch, Rohit Jain, Aram Vosoughi, G. Daniel Grass, Alice Yu, Wade J. Sexton, Scott M. Gilbert, Roger Li

**Affiliations:** Department of Genitourinary Oncology, H. Lee Moffitt Cancer Center, Tampa, FL 33612, USA

**Keywords:** radical cystectomy, adjuvant therapy, surgical margins, multi-modal therapy

## Abstract

Introduction and Objective: Muscle invasive bladder cancer with extravesical extension is an aggressive disease entity that requires multimodal therapy. The benefits of adjuvant chemotherapy (AC) in patients with a positive soft-tissue surgical margin (STSM), however, are relatively unknown due to exclusion of this population in randomized controlled trials of AC. We sought to define survival benefits in this patient population through our institutional bladder cancer database. Methods: Retrospective review of all patients undergoing radical cystectomy for urothelial carcinoma of the bladder from 2004–2020 with ≥pT3b disease irrespective of neoadjuvant chemotherapy (NAC) use was conducted. Progression-free survival (PFS) and overall survival (OS) estimates were obtained using the Kaplan-Meier method with log-rank test, and the Cox-proportional hazards model was used to identify predictors of improved PFS and OS. AC was defined by any chemotherapy use within 90 days of cystectomy, regardless of STSM status. Results: 476 patients with pT3b disease or worse were identified. Median follow-up was 12.3 months. An amount of 21% of patients were treated with AC. An amount of 24% of patients had positive STSM. Median OS for patients with positive STSM was 8.4 months [95% CI 7–11.5] and 18.3 months [95% CI 15.6–20.8] (*p* < 0.001) for patients with negative STSM. In the overall cohort, positive STSM (HR 1.93, 95% CI 1.45–2.57, *p* < 0.001), AC use (HR 0.68, 95% CI 0.51–0.90, *p* = 0.007), and pN1–3 disease (HR 1.47, 95% CI 1.16–1.87, *p* = 0.002) were independent predictors of OS when adjusted for performance status, pT-stage, and neoadjuvant chemotherapy use. In patients with positive STSM, median survival was seven months [95% CI 5.2–8.4] without AC, compared to 16.2 months [95% CI 11.5–52.5] with AC (*p* = 0.0038). For patients with negative STSM, median survival was 17.4 months [95% CI 14–20.1] without AC compared to 22.3 months [95% CI 17.2–36.9] with AC (*p* = 0.23). In patients with positive STSM, AC use was the only factor associated with an OS benefit with a HR of 0.41 (95% CI 0.21–0.78, *p* = 0.007). In patients with negative STSM, pT4 and pN1–3 disease were the only factors associated with worse overall survival with a HR of 1.32 (95% CI 1.00–1.74, *p* = 0.050) and 1.97 (95% CI 1.49–2.60, *p* < 0.001), respectively. Conclusions: Administration of adjuvant chemotherapy is of particular benefit in patients with positive STSM following radical cystectomy for gross extravesical disease. Positive STSM may be a representative of “early metastatic” or micrometastatic disease.

## 1. Introduction

A number of randomized clinical trials assessing adjuvant chemotherapy (AC) following radical cystectomy (RC) have been performed over the past four decades to delineate a potential survival benefit in patients with locally advanced disease [1]. While many of these evaluations were terminated early due to poor accrual or lack of benefit, meta-analysis data suggest a 6% five-year absolute survival benefit favoring adjuvant cisplatin-based chemotherapy (AC) [1]. Despite these benefits, nearly 40% of patients may experience a Grade 3 or worse adverse event while receiving AC, and more than half of patients may require dose reduction [2]. Notably, some patients who receive adjuvant systemic therapy may not derive a benefit at all, highlighting the ongoing need to identify features that may be associated with improved survival following adjuvant therapy [3,4,5,6].

Individuals with positive soft tissue surgical margins (STSM) at RC represent a unique subset of patients who may benefit from adjuvant treatments given their poor five-year overall survival rates below 30% [7,8]. Current recommendations for adjuvant systemic therapy following RC are based on pathologic T and N staging and prior neoadjuvant chemotherapy (NAC), with consideration of adding radiotherapy (XRT) when positive surgical margins are noted. Contemporary RCTs of AC, however, do not report the incidence of STSM or excluded patients with positive STSM from enrollment. Hence, the benefit of AC in this cohort of patients is not well defined [2,4,9,10].

Herein, we sought to identify the relationship between STSM and response to AC following RC. We focused on a subset of patients with pathologic gross extravesical disease ≥ pT3b given the higher likelihood of positive STSM in this clinical setting. We hypothesized that patients with positive STSM would derive greater survival benefit from AC than those whose primary cancers were completely extirpated [3,11].

## 2. Methods

Following Institutional Review Board approval, a retrospective review of 1954 patients undergoing RC at Moffitt Cancer Center from October 2004 to December 2020 was performed. Identification of patients who underwent RC with curative intent for primary urothelial carcinoma of the bladder and ≥pT3b disease on final pathologic assessment resulted in a cohort of 476 patients. Patients undergoing palliative cystectomy, en bloc cystectomy for non-urothelial malignancies, cystectomy for pure variant histology, for benign disease, or those without reported soft-tissue margin status were excluded. 

Baseline demographics and clinicopathologic features were recorded. Pre- and post-surgical systemic chemotherapy administration was reviewed. Curative intent RCs were performed by dedicated urologic oncologists, and extent of lymphadenectomy and organ sparing were determined based on pre-operative multidisciplinary consensus and intraoperative assessment. AC was defined as any post-surgical chemotherapy administered within 90 days following RC, regardless of STSM status, in the absence of clinical disease recurrence. Adjuvant radiotherapy was administered based on multidisciplinary consensus based on the risk of regional versus systemic progression. All specimens were reviewed by dedicated genitourinary pathologists, and microscopic or macroscopic presence of disease at any soft-tissue margin was considered positive. All patients received standard-of-care post-operative care with cross-sectional imaging for disease surveillance and biopsy of lesions suspicious for metastasis based on the treating physician’s discretion.

### Statistical Analysis

Continuous and categorical variables were compared using *t*-test and the chi-squared test, respectively. A progression-free survival (PFS) event was defined as the time to evidence of clinical disease progression, recurrence or all-cause mortality. Living patients were censored at the time of last known follow-up without disease progression/recurrence for PFS, and at last known follow up for overall survival (OS). PFS and OS estimates were calculated using the Kaplan–Meier (KM) method, and the log-rank test was used to compare PFS and OS when cohorts were compared. The cox-proportional hazards model was used to identify factors associated with PFS and OS. Models were adjusted a priori for age, pathologic T and N stage, ECOG performance status, NAC and AC use, and STSM status. Statistical tests were two-sided, significance was defined as *p*-value of <0.05, and analysis was performed using Jamovi version 2.3. 

## 3. Results

### 3.1. Demographic and Clinicopathologic Features

Baseline demographics stratified by STSM status are demonstrated in Table 1. Of the 476 patients, 112 (23.5%) had a positive STSM. These patients were more likely to have pT4, as opposed to pT3b disease, when compared to patients with negative STSM (73% vs. 46%, *p* < 0.001). No differences in the incidence of pre-operative renal insufficiency (41.1% positive STSM vs. 36.6% negative STSM, *p* = 0.2), receipt of NAC (46.4% positive STSM vs. 42.9% negative STSM, *p* = 0.51), or AC (19.8% positive STSM vs. 20.0% negative STSM, *p* = 0.77) were noted between the groups. An amount of 156/208 (75%) of patients receiving NAC received a cisplatin based regimen. Overall, 92 patients (19.3%) received AC. Demographics stratified by AC use are shown in Supplementary Table S1. A larger proportion of patients with pT4 disease was noted in the AC group, though this did not reach statistical significance (61% vs. 50%, *p* = 0.054).

### 3.2. Survival Estimates

**Figure 1:**
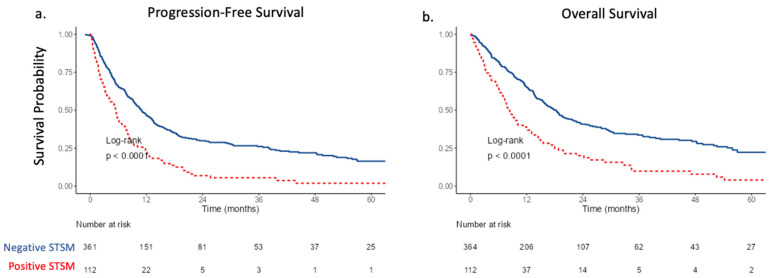
(**a**) Progression-free survival and (**b**) overall survival stratified by soft tissue surgical margin (STSM) status in patients undergoing radical cystectomy.

PFS and OS were significantly worse for patients with positive STSM compared to those with negative STSM (Figure 1). Median PFS for patients with positive and negative STSM were 5.4 months [95% CI 3.7–7.3] vs. 10.9 months [95% CI 9.4–13.2] (*p* < 0.001), respectively; OS was 8.4 months [95% CI 7–11.5] vs. 18.3 months [95% CI 15.6–20.8] (*p* < 0.001). Consistent with previous reports, AC conferred improved PFS (Median 13.2 months [95% CI 11.2–18.9] vs. 8.0 months [95% CI 6.8–9.9], *p* = 0.015) and OS (Median 19.8 months [95% CI 16–33.1] vs. 13.9 months [95% CI 12.2–17.4], *p* = 0.045) (Supplementary Figure S1). Multivariable Cox-proportional hazards model demonstrated positive STSM to be the strongest predictor of poor PFS (HR 2.04, 95% CI 1.55–2.68, *p* < 0.001) and OS (HR 1.93, 95% CI 1.45–2.57, *p* < 0.001) (Table 2). Additionally, positive nodal metastases (HR 1.47, 95% CI 1.16–1.87, *p* = 0.002) diminished OS, while the receipt of AC improved OS (HR 0.68, 95% CI 0.51–0.91, *p* = 0.009). 

**Figure 2:**
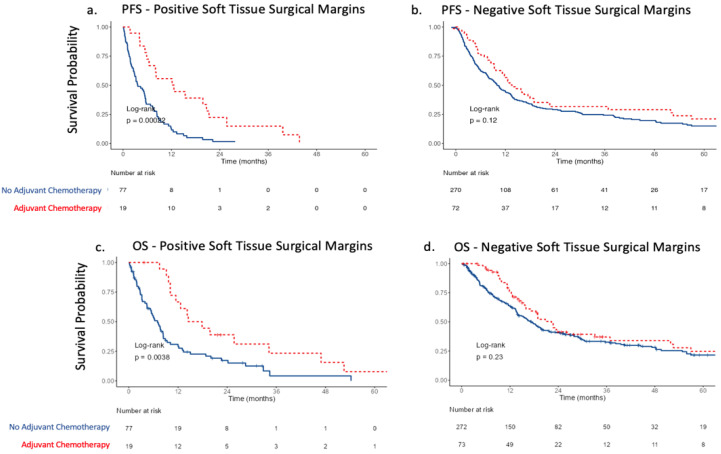
Progression-free survival stratified by adjuvant chemotherapy following radical cystectomy in (**a**) patients with positive soft tissue surgical margins and (**b**) patients with negative soft tissue surgical margins; overall survival stratified by adjuvant chemotherapy following radical cystectomy in (**c**) patients with positive soft tissue surgical margins and (**d**) patients with negative soft tissue surgical margins.

Interestingly, benefit in PFS derived from AC was only seen in patients with positive STSM (Median PFS 12.6 months [95% CI 6.5–25.7] vs. 3.7 months [95% CI 2.9–5.6], *p* < 0.001) (Figure 2a) and not in those with negative STSM (Median PFS 13.6 months [95% CI 11.2–18.9] vs. 10.0 months [95% CI 8–13], *p* = 0.12) (Figure 2b). Similarly, only those with positive STSM derived benefits in OS (Median OS 16.2 months [95% CI 11.5–52.5] vs. 7.0 months [95% CI 5.2–8.4], *p* = 0.004) compared to those with negative STSM (Median OS 22.3 months [95% CI 17.2–36.9] vs. 17.4 months [95% CI 14–20.1], *p* = 0.23) (Figure 2c,d). On Cox-proportional hazard models in patients with positive STSM, AC use was the only variable associated with OS benefit with a hazard ratio (HR) of 0.41 (95% CI 0.21–0.78, *p* = 0.007) when controlling for age, ECOG performance status, pathologic T and N stage, and NAC use (Supplementary Table S2). In contrast, for patients with negative STSM, pT4 disease (HR of 1.32, 95% CI 1.00–1.74, *p* = 0.050) and nodal metastases (HR 1.97, 95% CI 1.49–2.60, *p* < 0.001) were associated with worse overall survival, while AC was not (HR 0.74, 95% CI 0.53–1.03, *p* = 0.07) (Supplementary Table S3).

## 4. Discussion

It is well established that positive surgical margins following radical cystectomy and the presence of extravesical disease are independent predictors for poor overall survival [7,11]. Our cohort corroborates these findings, as positive STSM was the strongest independent predictor for poor survival in the entire cohort despite the potential rate of increased occult nodal metastasis in the positive STSM cohort. Interestingly, in patients with positive STSM, the traditional prognostic significance of the pathologic TNM system was lost, as pT (T3b vs. T4) and pN (N1–3 and Nx vs. N0) staging were not found to confer survival differences. These findings highlight the importance of achieving complete extirpation in locally advanced disease, as positive STSM trumps other prognostic variables. 

Guideline-based management specific to patients with positive STSM is limited to the use of adjuvant radiation to achieve local control [12]. The evidence supporting the use of adjuvant radiotherapy is derived from a Phase II randomized-controlled trial of sandwich chemotherapy-XRT-chemotherapy versus AC alone. Though patients in the combination arm derived a benefit in locoregional control at two years, a difference in disease-free survival or overall survival was not observed. Moreover, nearly 50% of patients had squamous cell carcinoma of the bladder, and patients with positive STSM were excluded from trial enrollment [13]. Importantly, no guidance is available regarding the use of AC in patients with positive STSM due to the dearth of evidence. While a recent meta-analysis using data from 10 randomized controlled trials suggests a 6% absolute survival benefit at five years, those trials utilizing contemporary chemotherapy regimens either excluded or did not report on positive STSM status [2,4,9,10,14,15]. 

From our study, an overall survival benefit from the use of AC was observed only in patients with a positive STSM. In contrast, no benefit was seen from the use of AC in patients with pT3b/4 disease and negative STSM. This discrepancy highlights the disease state in which systemic therapy may confer clinical benefit. While the benefit of AC following an R0 cystectomy is equivocal, there is a resounding survival advantage in patients with metastatic disease who received various systemic therapeutic regimens [16,17,18,19,20,21,22]. While trial data suggests median survival in patients receiving adjuvant therapy after an R0 resection is between four and six years, survival after receiving salvage therapy in the metastatic setting is between 11–16 months [1,16,18,20]. Our median survival of 16.2 months in patients with positive STSM receiving AC more closely represents survival in the metastatic setting. As such, patients with positive STSM due to locally advanced disease may represent an “early metastatic” state characterized by micrometastatic disease prevalence, which may benefit from adjuvant treatment. This concept was corroborated in the correlative circulating-tumor DNA (ctDNA) study within the IMvigor010 trial [6,23]. Adjuvant atezolizumab use was not associated with improved disease-free survival when compared to observation, and patients that were ctDNA positive on Cycle 1/Day 1 of therapy had poorer overall survival than patients that were ctDNA negative. Adjuvant atezolizumab, however, provided a survival benefit for patients that were ctDNA positive, while a difference in survival was not observed in the ctDNA negative cohort. 

These results have clear implications for future clinical trial design, strongly arguing for the inclusion of patients with positive STSM in future clinical trials based on ctDNA assays defining patients in the “molecularly metastatic” state. Patients with ctDNA positivity may represent a cohort with a higher risk of disease recurrence as defined by conventional criteria, and utilizing such assays as an enrollment criterium for clinical trials may significantly decrease the number of patients needed to be enrolled due to increases in event rates. In addition, experimental therapy versus standard of care evaluations can be performed in ctDNA positive groups, potentially identifying a subset of patients that are most likely to benefit from novel therapies while minimizing the burden of adverse events. Additionally, ctDNA clearance may represent freedom disease, and if utilized as a surrogate for survival, could drastically shorten the duration of clinical trials. 

Finally, our findings that AC conferred survival advantage in patients with positive STSM are counter to those reported by Neuzillet et al. [24]. In a multi-variable analysis of 154 chemo-naïve patients undergoing RC and 154 matched controls, they found that AC did not confer a survival benefit in the overall cohort. Notably, 19% (29/154) of patients in this study had organ confined disease (≤pT2), and 34% of these patients (10/29) had positive STSM. Therefore, the benefit of AC in this group may have been dampened by the inclusion of patients with fundamentally less aggressive disease and those with the absence of micrometastatic disease.

The retrospective nature of our analysis produces several limitations. Consistent with other observational data, unmeasured confounders relating to patient fitness and comorbidity status following RC were unable to be captured, and these features may have influenced AC administration. Despite this, no significant differences were encountered in the available demographic and pathologic features between the groups that did and did not receive AC or based on margin status. In addition, the definition of AC in our study was not exclusive to a particular regimen or duration of therapy. Though the regimens were heterogeneous, our findings may be more applicable to larger populations that may not otherwise qualify for a single type or interval of therapy, especially given the frequent dose reductions and challenges pertaining to AC administration encountered in the adjuvant setting. In addition, of 23 patients receiving adjuvant XRT, only six patients received independent treatment without concomitant AC, so we were unable to determine the effects of multimodal adjuvant local plus systemic therapy. 

Despite these limitations, our findings are consistent with results from meta-analysis and large observational datasets regarding the benefit of AC in patients with locally advanced disease following RC. Moreover, they highlight a subgroup of patients with poor survival following RC due to positive STSM who may derive even greater benefit from AC administration, as positive STSM may be a harbinger of “early metastatic” or micrometastatic disease. 

## 5. Conclusions

Patients with positive soft tissue surgical margins following radical cystectomy for locally advanced urothelial cancer have poorer survival than patients with negative margins. Adjuvant chemotherapy prolongs progression-free and overall survival in patients with gross extravesical disease. The presence of positive soft tissue margins following cystectomy may be a harbinger of “early metastatic” or micrometastatic disease, highlighting a cohort that may derive particular benefit from adjuvant chemotherapy.

## Figures and Tables

**Table 1:** Clinicodemographic Features by Soft Tissue Margin Status.

	Negative (*N* = 364)	Positive (*N* = 112)	Total (*N* = 476)	*p* Value
Age				0.264
Mean (SD)	70.5 (10.6)	71.7 (10.0)	70.8 (10.5)	
Range	36.0–92.0	33.0–92.0	33.0–92.0	
Sex				0.783
Female	80 (22.0%)	26 (23.2%)	106 (22.3%)	
Male	284 (78.0%)	86 (76.8%)	370 (77.7%)	
pT Stage				<0.001
T3b	197 (54.1%)	30 (26.8%)	227 (47.7%)	
T4	167 (45.9%)	82 (73.2%)	249 (52.3%)	
pN Stage				<0.001
Nx	15 (4.1%)	16 (14.2%)	31 (6.5%)	
N0	203 (55.8%)	48 (42.9%)	251 (52.7%)	
N1–3	146 (40.1%)	48 (42.9%)	194 (40.8%)	
ECOG				0.086
Missing	1 (0.3%)	0 (0%)	1 (0.2%)	
0	253 (69.5%)	66 (58.9%)	319 (67.0%)	
1	84 (23.1%)	29 (25.9%)	113 (23.8%)	
2	18 (4.9%)	11 (9.8%)	29 (6.1%)	
3	6 (1.6%)	5 (4.5%)	11 (2.3%)	
4	2 (0.6%)	1 (0.9%)	3 (0.6%)	
Neoadjuvant Chemotherapy				0.505
No	208 (57.1%)	60 (53.6%)	268 (56.3%)	
Yes	156 (42.9%)	52 (46.4%)	208 (43.7%)	
Adjuvant Chemotherapy				0.771
N-Miss	19 (5.2%)	16 (14.3%)	35 (7.4%)	
No	272 (74.8%)	77 (80.2%)	349 (73.3%)	
Yes	73 (20.0%)	19 (19.8%)	92 (19.3%)	
≥CKD3				0.197
N-Miss	54 (14.8%)	21 (18.7%)	75 (15.8%)	
No	177 (48.6%)	45 (40.2%)	222 (46.6%)	
Yes	133 (36.6%)	46 (41.1%)	179 (37.6%)	

**Table 2:** Univariable and Multivariable Cox-Proportional Hazards Model for Progrssion-Free Survival and Overall Survival in All Patients.

			Progression-Free Survival		Overall Survival	
		*N*	HR (Univariable)	HR (Multivariable)	HR (Univariable)	HR (Multivariable)
Age	Mean (SD)	70.8 (10.5)	1.01 (1.00–1.02, *p* = 0.086)	1.01 (1.00–1.02, *p* = 0.118)	1.01 (1.00–1.02, *p* = 0.140)	1.01 (0.99–1.02, *p* = 0.304)
ECOG						
	0	292 (66.4)	-	-	-	-
	1	106 (24.1)	1.15 (0.89–1.47, *p* = 0.289)	1.07 (0.83–1.38, *p* = 0.603)	1.15 (0.88–1.51, *p* = 0.297)	1.10 (0.84–1.44, *p* = 0.504)
	2	29 (6.6)	1.64 (1.08–2.49, *p* = 0.020)	1.54 (1.01–2.34, *p* = 0.046)	1.50 (0.97–2.34, *p* = 0.071)	1.30 (0.83–2.04, *p* = 0.252)
	3	10 (2.3)	2.08 (1.07–4.06, *p* = 0.032)	1.40 (0.70–2.79, *p* = 0.340)	1.58 (0.78–3.21, *p* = 0.207)	1.15 (0.56–2.40, *p* = 0.700)
	4	3 (0.7)	1.70 (0.42–6.84, *p* = 0.457)	1.90 (0.47–7.72, *p* = 0.372)	2.97 (0.73–11.99, *p* = 0.127)	3.52 (0.86–14.44, *p* = 0.080)
Positive Soft Tissue Margin						
	No	344 (78.2)	-	-	-	-
	Yes	96 (21.8)	2.19 (1.70–2.83, *p* < 0.001)	2.04 (1.55–2.68, *p* < 0.001)	2.14 (1.65–2.79, *p* < 0.001)	1.93 (1.45–2.57, *p* < 0.001)
pT Stage						
	T3b	212 (48.2)	-	-	-	-
	T4	228 (51.8)	1.40 (1.13–1.73, *p* = 0.002)	1.19 (0.94–1.50, *p* = 0.145)	1.43 (1.14–1.79, *p* = 0.002)	1.21 (0.95–1.55, *p* = 0.128)
pN Stage						
	N0	230 (52.3)	-	-	-	-
	N1–3	184 (41.8)	2.12 (1.70–2.65, *p* < 0.001)	2.16 (1.73–2.72, *p* < 0.001)	1.56 (1.23–1.97, *p* < 0.001)	1.47 (1.16–1.87, *p* = 0.002)
	Nx	26 (5.9)	1.80 (1.15–2.81, *p* = 0.011)	1.42 (0.89–2.25, *p* = 0.137)	1.90 (1.20–3.01, *p* = 0.006)	1.55 (0.96–2.51, *p* = 0.076)
Neoadjuvant Chemotherapy						
	No	245 (55.7)	-	-	-	-
	Yes	195 (44.3)	1.14 (0.92–1.41, *p* = 0.219)	1.12 (0.89–1.40, *p* = 0.330)	1.02 (0.81–1.29, *p* = 0.839)	0.94 (0.74–1.20, *p* = 0.626)
Adjuvant Chemotherapy						
	No	349 (79.3)	-	-	-	-
	Yes	91 (20.7)	0.71 (0.55–0.93, *p* = 0.012)	0.59 (0.45–0.77, *p* < 0.001)	0.76 (0.57–1.00, *p* = 0.052)	0.68 (0.51–0.91, *p* = 0.009)

## Data Availability

The data presented in this study are available on request from the corresponding author. The data are not publicly available due to ethical considerations regarding an institutional dataset in a vulnerable patient population.

## Supplementary Materials

The following supporting information can be downloaded at: https://www.mdpi.com/article/10.3390/curroncol30030245/s1, Figure S1: (a) Progression-Free and (b) Overall survival stratified by Adjuvant Chemotherapy status in patients undergoing radical cystectomy; Table S1: Clinicodemographic Features by Adjuvant Chemotherapy Status; Table S2: Univariable and Multivariable Cox-Proportional Hazards Model for Progression-Free Survival and Overall Survival Patients with Positive Surgical Margins; Table S3: Univariable and Multivariable Cox-Proportional Hazards Model for Progression-Free Survival and Overall Survival Patients with Negative Surgical Margins
