# Supplementary material for: Survival Benefits of Adjuvant Chemotherapy for Positive Soft Tissue Surgical Margins Following Radical Cystectomy in Bladder Cancer with Extravesical Extension
Source: Curr Oncol. 2023 Mar 10;30(3):3223–31. doi: 10.3390/curroncol30030245 (PMC10046994; doi:10.3390/curroncol30030245)
Supplement: Supplementary file 1 [file curroncol-30-00245-s001.zip › curroncol-2242894-supplementary.pdf]

Supplementary Table S1 – Clinicodemographic Features by Adjuvant Chemotherapy Status

	No AC (N=349)	AC (N=92)	Total (N=441)	p value
**Age**				0.076
Mean (SD)	70.9 (11.8)	68.5 (9.5)	70.4 (11.4)	
**Sex**				0.046
Female	83 (23.8%)	13 (14.1%)	96 (21.8%)	
Male	266 (76.2%)	79 (85.9%)	345 (78.2%)	
**pT Stage**				0.054
T3b	176 (50.4%)	36 (39.1%)	212 (48.1%)	
T4	173 (49.6%)	56 (60.9%)	229 (51.9%)	
**pN Stage**				0.209
Nx	23 (6.6%)	3 (3.3%)	26 (5.9%)	
N0	186 (53.3%)	44 (47.8%)	230 (52.2%)	
N1-3	140 (40.1%)	45 (48.9%)	185 (42.0%)	
**ECOG**				0.930
N-Miss	0	1 (1.1%)	1 (0.2%)	
0	229 (65.6%)	63 (68.5%)	292 (66.4%)	
1	86 (24.6%)	20 (21.7%)	106 (24.1%)	
2	24 (6.9%)	5 (5.4%)	29 (6.6%)	
3	8 (2.3%)	2 (2.2%)	10 (2.2%)	
4	2 (0.6%)	1 (1.1%)	3 (0.7%)	
**Neoadjuvant Chemotherapy**				0.834
No	193 (55.3%)	52 (56.5%)	245 (55.6%)	
Yes	156 (44.7%)	40 (43.5%)	196 (44.4%)	
**Soft Tissue Margin**				0.771
Negative	272 (77.9%)	73 (79.3%)	345 (78.2%)	
Positive	77 (22.1%)	19 (20.7%)	96 (21.8%)	

Supplementary Table S2 - Univariable and Multivariable Cox-Proportional Hazards Model for Progression-Free Survival and Overall Survival Patients with Positive Surgical Margins

		N	Progression-Free Survival HR (univariable)	Progression-Free Survival HR (multivariable)	Overall Survival HR (univariable)	Overall Survival HR (multivariable)
Age	Mean (SD)	72.0 (9.5)	1.01 (0.99-1.04, p=0.406)	1.00 (0.97-1.03, p=0.908)	1.01 (0.99-1.04, p=0.305)	1.00 (0.97-1.03, p=0.852)
ECOG						
	0	53 (55.2)	-	-	-	-
	1	27 (28.1)	0.96 (0.59-1.57, p=0.864)	1.11 (0.67-1.86, p=0.682)	1.02 (0.61-1.71, p=0.941)	1.02 (0.60-1.74, p=0.945)
	2	11 (11.5)	1.19 (0.58-2.45, p=0.642)	1.92 (0.88-4.18, p=0.101)	0.84 (0.39-1.81, p=0.661)	0.85 (0.38-1.90, p=0.687)
	3	4 (4.2)	1.37 (0.49-3.84, p=0.549)	0.95 (0.31-2.89, p=0.927)	0.79 (0.24-2.57, p=0.693)	0.61 (0.18-2.08, p=0.430)

Supplementary Table S2 - Univariable and Multivariable Cox-Proportional Hazards Model for Progression-Free Survival and Overall Survival Patients with Positive Surgical Margins

		N	Progression-Free Survival HR (univariable)	Progression-Free Survival HR (multivariable)	Overall Survival HR (univariable)	Overall Survival HR (multivariable)
	4	1 (1.0)	3.09 (0.41-23.01, p=0.271)	2.36 (0.31-18.16, p=0.410)	5.49 (0.72-41.97, p=0.101)	5.82 (0.72-46.92, p=0.098)
pT Stage						
	T3b	26 (27.1)	-	-	-	-
	T4	70 (72.9)	0.73 (0.45-1.19, p=0.203)	0.80 (0.47-1.35, p=0.406)	0.85 (0.51-1.40, p=0.515)	1.02 (0.59-1.75, p=0.952)
pN Stage						
	N0	42 (43.8)	-	-	-	-
	N1-3	41 (42.7)	1.89 (1.18-3.02, p=0.008)	2.01 (1.22-3.30, p=0.006)	1.20 (0.74-1.95, p=0.453)	1.06 (0.63-1.77, p=0.828)
	Nx	13 (13.5)	1.88 (0.91-3.87, p=0.089)	1.47 (0.68-3.19, p=0.327)	1.72 (0.81-3.66, p=0.156)	1.47 (0.65-3.33, p=0.360)
Neoadjuvant Chemotherapy						
	No	47 (49.0)	-	-	-	-
	Yes	49 (51.0)	1.00 (0.65-1.54, p=0.994)	0.85 (0.52-1.39, p=0.514)	0.87 (0.55-1.37, p=0.547)	0.73 (0.44-1.24, p=0.245)
Adjuvant Chemotherapy						
	No	77 (80.2)	-	-	-	-
	Yes	19 (19.8)	0.35 (0.19-0.62, p<0.001)	0.31 (0.16-0.61, p=0.001)	0.44 (0.24-0.78, p=0.005)	0.41 (0.21-0.78, p=0.007)

Supplementary Table S3 - Univariable and Multivariable Cox-Proportional Hazards Model for Progression-Free Survival and Overall Survival Patients with Negative Surgical Margins

		N	Progression-Free Survival HR (univariable)	Progression-Free Survival HR (multivariable)	Overall Survival HR (univariable)	Overall Survival HR (multivariable)
Age	Mean (SD)	70.4 (10.7)	1.01 (1.00-1.02, p=0.148)	1.01 (1.00-1.02, p=0.094)	1.01 (0.99-1.02, p=0.295)	1.01 (0.99-1.02, p=0.341)
ECOG						
	0	239 (69.5)	-	-	-	-
	1	79 (23.0)	1.10 (0.82-1.48, p=0.520)	1.07 (0.79-1.45, p=0.641)	1.11 (0.80-1.53, p=0.528)	1.09 (0.79-1.51, p=0.587)
	2	18 (5.2)	1.60 (0.96-2.68, p=0.072)	1.61 (0.95-2.73, p=0.077)	1.58 (0.91-2.73, p=0.106)	1.65 (0.95-2.89, p=0.078)
	3	6 (1.7)	2.08 (0.85-5.06, p=0.108)	2.25 (0.91-5.59, p=0.080)	1.84 (0.75-4.51, p=0.180)	1.89 (0.76-4.72, p=0.170)

Supplementary Table S3 - Univariable and Multivariable Cox-Proportional Hazards Model for Progression-Free Survival and Overall Survival Patients with Negative Surgical Margins

		N	Progression-Free Survival HR (univariable)	Progression-Free Survival HR (multivariable)	Overall Survival HR (univariable)	Overall Survival HR (multivariable)
	4	2 (0.6)	1.14 (0.16-8.13, p=0.898)	2.11 (0.29-15.42, p=0.461)	2.04 (0.28-14.64, p=0.478)	3.28 (0.45-24.15, p=0.243)
pT Stage						
	T3b	186 (54.1)	-	-	-	-
	T4	158 (45.9)	1.35 (1.06-1.73, p=0.015)	1.30 (1.01-1.69, p=0.044)	1.38 (1.06-1.79, p=0.017)	1.32 (1.00-1.74, p=0.050)
pN Stage						
	N0	188 (54.7)	-	-	-	-
	N1-3	143 (41.6)	2.47 (1.92-3.19, p<0.001)	2.50 (1.93-3.26, p<0.001)	1.98 (1.51-2.60, p<0.001)	1.97 (1.49-2.60, p<0.001)
	Nx	13 (3.8)	1.52 (0.84-2.77, p=0.167)	1.35 (0.73-2.51, p=0.345)	1.75 (0.96-3.19, p=0.068)	1.59 (0.85-2.96, p=0.144)
Neoadjuvant Chemotherapy						
	No	198 (57.6)	-	-	-	-
	Yes	146 (42.4)	1.12 (0.87-1.43, p=0.374)	1.09 (0.84-1.42, p=0.516)	1.02 (0.78-1.33, p=0.900)	1.00 (0.75-1.32, p=0.984)
Adjuvant Chemotherapy						
	No	272 (79.1)	-	-	-	-
	Yes	72 (20.9)	0.77 (0.57-1.05, p=0.096)	0.65 (0.47-0.88, p=0.006)	0.83 (0.60-1.14, p=0.249)	0.74 (0.53-1.03, p=0.074)

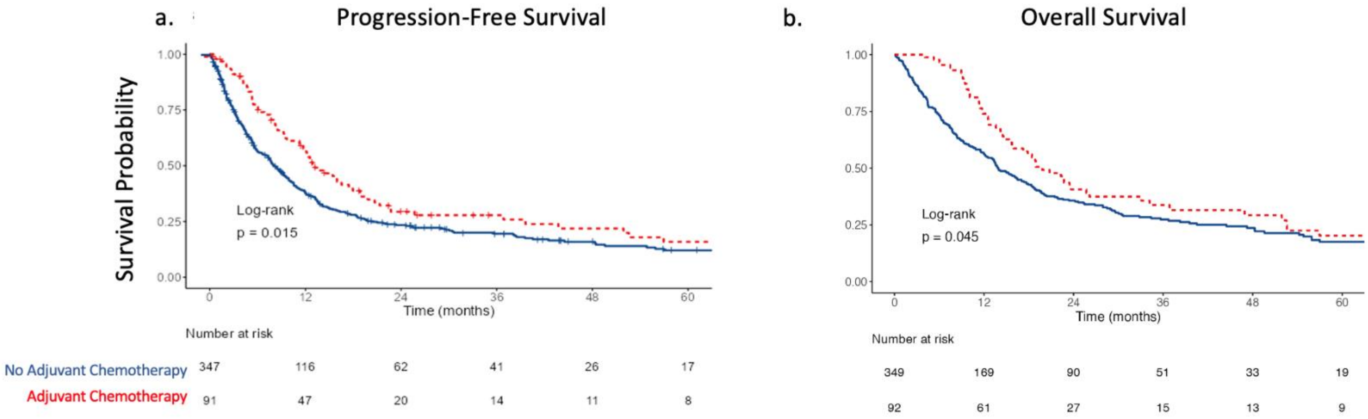

Supplementary Figure S1 - (a.) Progression-Free and (b.) Overall survival stratified by Adjuvant Chemotherapy status in patients undergoing radical cystectomy.
